# The underlying dimensions of DSM-5 PTSD symptoms and their relations with anxiety and depression in a sample of adolescents exposed to an explosion accident

**DOI:** 10.1080/20008198.2016.1272789

**Published:** 2017-02-08

**Authors:** Haibo Yang, Li Wang, Chengqi Cao, Xing Cao, Ruojiao Fang, Jianxin Zhang, Jon D. Elhai

**Affiliations:** ^a^ Laboratory for Traumatic Stress Studies, CAS Key Laboratory of Mental Health, Institute of Psychology, Chinese Academy of Sciences, Beijing, China; ^b^ Academy of Psychology and Behavior, Tianjin Normal University, Tianjin, China; ^c^ University of Chinese Academy of Sciences, Beijing, China; ^d^ Department of Psychology, and Department of Psychiatry, University of Toledo, Toledo, OH, USA

**Keywords:** Confirmatory factor analysis, DSM-5, PTSD, adolescents, man-made disaster, China

## Abstract

**Background:** A large number of empirical studies pertaining to the latent dimensions of DSM-5 PTSD symptoms have accumulated. However, there is still a lack of studies specific to youths.

**Objective:** This study sought to investigate the latent dimensions of DSM-5 PTSD symptoms in a sample of adolescents exposed to an explosion accident.

**Method:** Participants were 836 students (407 females and 428 males). Self-reported measures including the PTSD Checklist for DSM-5 and the anxiety and depression subscales of the 21-item Depression Anxiety Stress Scale were administered to participants. Confirmatory factor analysis (CFA) was implemented to test competing factor models.

**Results:** A seven-factor model composed of intrusion, avoidance, negative affect, anhedonia, externalizing behaviours, anxious arousal and dysphoric arousal factors emerged as the best fitting model, and PTSD’s factors displayed distinguishable correlations with external measures of anxiety and depression.

**Conclusions:** The findings provide and extend empirical evidence supporting the newly refined seven-factor hybrid model of DSM-5 PTSD symptoms, and have implications for further trauma-related clinical practice and research.

## 1. Introduction

Posttraumatic stress disorder (PTSD) is a debilitating psychiatric syndrome caused by exposure to traumatic stressors. According to the most recent revision of the *Diagnostic and Statistical Manual of Mental Disorders* (DSM-5; American Psychiatric Association, 2013), the criteria for PTSD are now composed of 20 symptoms which are further grouped into four clusters including intrusion (Criterion B), avoidance (Criterion C), negative alterations in cognitions and mood (Criterion D) and alterations in arousal and reactivity (Criterion E). The current four-factor conceptualization is constructed largely based on previous confirmatory factor analysis (CFA) studies on DSM-IV PTSD symptoms (Friedman, 2013). However, during the past three years, several theoretically and empirically driven alternatives were proposed that empirically challenged the DSM-5 four-factor model (c.f. Armour, Műllerová, & Elhai, 2016). Ongoing examination of the latent dimensions of PTSD is pertinent in refining clinically useful diagnostic procedures, developing sophisticated intervention programmes, and elucidating the underlying psychopathological and biological mechanisms of this disorder. In the current study, we investigated the latent dimensions of DSM-5 PTSD symptoms in a sample of adolescents recently exposed to an explosion accident, using these newer models.

Shortly after the release of the DSM-5, two six-factor alternative models of PTSD were almost simultaneously proposed by two independent research groups. The models included the anhedonia model (Liu et al., 2014) and the externalizing behaviours model (Tsai et al., 2015), and both were built on the basis of the latest development in the literature on the factor structure of DSM-IV PTSD symptoms, suggesting that PTSD’s hyperarousal symptoms should be further differentiated as two factors: dysphoric arousal and anxious arousal (c.f., Armour et al., 2015, 2016; Armour, 2015). The anhedonia model consists of intrusion, avoidance, negative affect, anhedonia, anxious arousal and dysphoric arousal factors, and rests on separating the current Criterion D symptoms into negative affect factor composed of symptoms involving enhanced negative affect/general distress (e.g., pervasive negative emotional state and negative beliefs) and anhedonia factor composed of symptoms involving reduced positive affect/anhedonia (e.g., inability to experience positive emotions and lack of interest). The distinction of negative affect and anhedonia has been supported by substantial empirical and theoretical studies (e.g., Cuthbert, 2014; Watson, 2009). The externalizing behaviours model is comprised of intrusion, avoidance, negative alterations in cognitions and mood, externalizing behaviours, anxious arousal and dysphoric arousal factors, and hinges on specifying a distinct externalizing behaviours factor. The externalizing behaviours factor is composed of irritable or aggressive behaviour (E1) and reckless or self-destructive behaviour (E2) which are typical externalizing symptoms representing deficits in emotion regulation and impulse (e.g., Friedman, 2013), and accordingly could be theoretically distinguished from other internally based PTSD symptoms (Tsai et al., 2015). Both of the six-factor models were found to outperform the DSM-5 four-factor model and other competing models in an epidemiological sample of Chinese earthquake survivors (Liu et al., 2014) and in a representative sample of US veterans (Tsai et al., 2015), respectively.

More recently, a seven-factor hybrid model which integrated the key elements of both six-factor models was proposed, and demonstrated superiority relative to the DSM-5 four-factor model and two six-factor models in a sample of US veterans and a sample of trauma-exposed undergraduates (Armour et al., 2015). The seven-factor hybrid model has received empirical support in subsequent CFA studies with adult samples exposed to various traumatic events (Seligowski & Orcutt, 2016), military-related trauma (Bovin et al., 2016) and typhoon (Mordeno, Carpio, Nalipay, & Saavedra, in press); and with adolescent samples exposed to earthquake (Wang et al., 2015) and various traumatic events (Liu, Wang, Cao, Qing, & Armour, 2016).

It should be noted that although the DSM-5 diagnostic criteria for PTSD are the same for youths and adults, developmental differences in posttraumatic symptoms have long been discussed (Helpman et al., 2015). Given that extant studies on the latent dimensions of DSM-5 PTSD symptoms were conducted mainly with adults, there is particular need for further studies with traumatized youths as relevant knowledge is relatively insufficient. To our knowledge, the newly refined seven-factor hybrid model was only validated in two studies with traumatized youths, and the types of trauma may moderate victims’ traumatic responses (e.g., Kelley, Weathers, McDevitt-Murphy, Eakin, & Flood, 2009). Additional studies with youths exposed to different traumatic events are needed. Moreover, validating a diagnostic model cannot merely depend on CFA fit statistics (e.g., Armour et al., 2016), and thereby evaluating the external convergent and discriminant validity of the model also is needed. In so doing, the current study investigated the latent dimensions of DSM-5 PTSD symptoms in a sample of adolescents recently exposed to an explosion accident. Using a CFA approach, we first tested four competing models including the current DSM-5 model, the anhedonia model, the externalizing behaviours model, and the seven-factor hybrid model (see Table 1 for symptom mappings). On the basis of previous CFA studies on traumatized youths (Liu et al., 2016; Wang et al., 2015), it was hypothesized that the seven-factor hybrid model would significantly outperform the other competing models in the current sample. Subsequently, the relationships between PTSD symptom factors and external measures of anxiety and depression were examined. According to previous theoretical and empirical studies suggesting that intrusion, avoidance and anxious arousal are typical anxiety-related rather than depression-related constructs (e.g., Armour et al., 2012; Elhai et al., 2011; Wang et al., 2013; Watson, 2009), we hypothesized that these anxiety-related PTSD constructs would associate more strongly with external measures of anxiety than depression. Based on previous theoretical and empirical studies suggesting that anhedonia is a typical depression-related rather than anxiety-related construct (Watson, 2009), we hypothesized that this depression-related PTSD construct would associate more strongly with external measures of depression than anxiety. Finally, built on previous theoretical and empirical studies suggesting negative affect and dysphoric arousal are involving both anxiety and depression-related symptoms (e.g., Elhai et al., 2011; Wang et al., 2013; Watson, 2009), we hypothesized that the two both anxiety and depression-related PTSD constructs would associate with depression and anxiety equivalently.

**Table 1.** Symptom mappings for confirmatory factor analysis.

PTSD symptoms	Model 1 (DSM-5)	Model 2 (Externalizing behaviours)	Model 3 (Anhedonia)	Model 4 (Hybrid)
B1. Intrusive thoughts	In	In	In	In
B2. Nightmares	In	In	In	In
B3. Flashbacks	In	In	In	In
B4. Emotional cue reactivity	In	In	In	In
B5. Physiological cue reactivity	In	In	In	In
C1. Avoidance of thoughts	Av	Av	Av	Av
C2. Avoidance of reminders	Av	Av	Av	Av
D1. Trauma-related amnesia	NACM	NACM	NA	NA
D2. Negative beliefs	NACM	NACM	NA	NA
D3. Distorted blame	NACM	NACM	NA	NA
D4. Pervasive negative emotional state	NACM	NACM	NA	NA
D5. Lack of interest	NACM	NACM	An	An
D6. Feeling detached	NACM	NACM	An	An
D7. Inability to experience positive emotions	NACM	NACM	An	An
E1. Irritability/aggression	Hy	EB	DA	EB
E2. Recklessness	Hy	EB	DA	EB
E3. Hypervigilance	Hy	AA	AA	AA
E4. Exaggerated startle	Hy	AA	AA	AA
E5. Difficulty concentrating	Hy	DA	DA	DA
E6. Sleep disturbance	Hy	DA	DA	DA

PTSD = Posttraumatic stress disorder. Model 1 = the DSM-5 model; Model 2 = the externalizing behaviours model; Model 3 = the anhedonia model; Model 4 = the seven-factor hybrid model. In = Intrusion; Av = Avoidance; NACM = Negative alterations in cognitions and mood; Hy = Hyperarousal; EB = Externalizing behaviours; AA = Anxious arousal; DA = Dysphoric arousal; NA = Negative affect; An = Anhedonia.

## 2. Method

### 2.1. Procedure and participants

On 12 August 2015, a series of explosions caused by chemicals hit the Binhai New Area of Tianjin, China. The explosion accident was China’s worst industrial disaster in years, which killed 173 people, injured 797 others, and destroyed or damaged hundreds of buildings.

This study was conducted approximately three months after the disaster. The sample was recruited from a primary school and a middle school located nearest to the blast site. A total of 1242 students who were at or above third grade level and presented at the school participated in this study. Written informed consent was obtained from each child and their guardian. Group testing was conducted in the schools with monitoring by trained research assistants and school teachers. The study protocol was approved by the Institutional Review Board of the Institute of Psychology, Chinese Academy of Sciences.

Among the initial participants, 406 students who reported not personally experiencing the disaster in local (i.e., did not live in the communities near the site of explosions during the accident) were excluded from analyses. Accordingly, the final effective sample was comprised of 836 students (407 females and 428 males) with a mean age of 12.5 years (*SD* = 2.3, range: 9–17). Of the participants, 791 (94.6%) were self-reported as Han ethnicity, and 41 (4.4%) were other ethnicities in China (including Hui, Man, Mongolian, etc.). During the accident, 512 (61.2%) participants witnessed the explosions, 433 (51.8%) smelled a pungent related odour, 50 (6.0%) were trapped in a house or somewhere, 84 (10%) were injured, 61 (7.3%) witnessed a death of someone, 135 (16.1%) witnessed someone being hurt, 3 (0.4%) lost at least one of their family members and 38 (4.5%) lost at least one of their schoolmates or friends.

### 2.2. Measures

PTSD symptoms were measured using the PTSD Checklist for DSM-5 (PCL-5, Weathers, 2013). The PCL-5 is a 20-item self-reported measure constructed on the basis of DSM-5 diagnostic criteria, and each item is rated on a 5-point Likert scale (0 = *not at all* to 4 = *extremely*). The psychometrics of the PCL-5 has been demonstrated (e.g., Bovin et al., 2016). The Chinese version of the PCL-5 was adapted via a two-stage process of translation and reverse translation, and has been used in traumatized Chinese youths (Liu et al., 2016; Wang et al., 2015). In this study, the PCL-5 items were completed regarding the Tianjin explosions, and Cronbach’s α was .93 for the scale in the present sample.

Anxiety and depression symptoms were measured with the corresponding subscales of the 21-item Depression Anxiety Stress Scale (DASS-21, Lovibond & Lovibond, 1995). Each subscale includes seven items scoring on a 4-point Likert scale (0 = *did not apply to me at all* to 3 = *applied to me very much of the time*). The reliability and validity of the DASS-21 has been well documented (e.g., Szabó, 2010). The Chinese version of the DASS-21 has been validated and widely used in Chinese populations (e.g., Wang et al., 2016). In the present sample, Cronbach’s αs were .80 and .81 for the anxiety and depression subscales, respectively.

### 2.3. Data analysis

Among the participants, 25 (3.0%) were missing one or two PCL-5 items, 16 (1.9%) missing one anxiety item and 14 (1.7%) missing one depression items. Missing data were handled with full information maximum likelihood estimation. CFAs were conducted with Mplus Version 7.0. First, maximum likelihood estimation with a mean-adjusted, scale Satorra-Bentler chi-square (S-B χ^2^) was implemented to estimate model parameters for four PTSD models. Overall model fit was evaluated with the comparative fit index (CFI), the Tucker–Lewis index (TLI), the root-mean square error of approximation (RMSEA) and the standardized root mean square residual (SRMR). CFI and TLI value ≥ .95/.90, RMSEA ≤ .06/.08, and SRMR ≤ .08 indicate an excellent/acceptable fit (Kline, 2011). The corrected scaled χ^2^ difference test and the Bayesian information criterion (BIC) were used to compare nested and non-nested models, respectively. A lower BIC value of at least 10 evidences a better fitting model (Raftery, 1995). Second, a nine-factor model comprised by seven PTSD factors and two additional anxiety and depression factors was specified to test. DASS-21 items were treated as ordinal variables rather than continuous variables as they have fewer than five response options (Wirth & Edwards, 2007). Accordingly, weighted least squares estimation with a mean- and variance-adjusted chi-square (WLSMV) was used in CFA. Based on the nine-factor model, Wald chi-square tests of parameter constraints to test the null hypothesis that the difference between pairs of correlations is zero were implemented to assess hypothesized relationships between PTSD’s factors and external anxiety and depression.

## 3. Results

The mean PCL-5 score for the current sample was 9.2 (*SD* = 11.7, range: 0–80), the median was 5, and 37 (4.4%) participants were screened as probable PTSD cases based on the DSM-5 diagnostic algorithm of at least one intrusion symptom, one avoidance symptom, two negative alterations in cognitions and mood symptoms and two arousal symptoms endorsed as 2 or greater. The mean scores on DASS-21 anxiety and depression subscales were 2.4 (*SD* = 3.3, range: 0–21) and 1.7 (*SD* = 2.9, range: 0–19), and the medians were both 1.

All four PTSD models demonstrated excellent fit (see Table 2 for model goodness of fit indices). Results from χ^2^ difference tests indicated that: the DSM-5 model fit significantly poorer than the externalizing behaviours model (Δχ^2^ (9) = 72.22, *p* < .01), the anhedonia model (Δχ^2^ (9) = 78.36, *p* < .01), and the seven-factor hybrid model (Δχ^2^ (15) = 96.89, *p* < .01); the seven-factor hybrid model fit significantly better than the externalizing behaviours model (Δχ^2^ (6) = 28.87, *p* < .01) as well as the anhedonia model (Δχ^2^ (6) = 12.90, *p* < .05). Regarding non-nested models comparison, the anhedonia model fit better than the externalizing behaviours model evidenced by a ∆BIC of 54.937. Therefore, the seven-factor hybrid model was selected as the best fitting model.

**Table 2.** Model goodness of fit indices.

Models	S-B χ^2^	*df*	CFI	TLI	SRMR	RMSEA	RMSEA 90% CI	BIC
Model 1	308.965	164	.952	.945	.049	.033	.027–.038	35198.973
Model 2	239.743	155	.972	.966	.045	.026	.019–.032	35092.284
Model 3	220.015	155	.979	.974	.040	.022	.015–.029	35037.347
Model 4	207.493	149	.981	.975	.039	.022	.014–.028	35049.122

*N* = 836. Model 1 = the DSM-5 model; Model 2 = the externalizing behaviours model; Model 3 = the anhedonia model; Model 4 = the seven-factor hybrid model. CFI = Comparative fit index; TLI = Tucker-Lewis index; SRMR = Standardized root mean square residual; RMSEA = Root mean square error of approximation; CI = Confidence interval; BIC = Bayesian information criterion.

**Figure 1.**
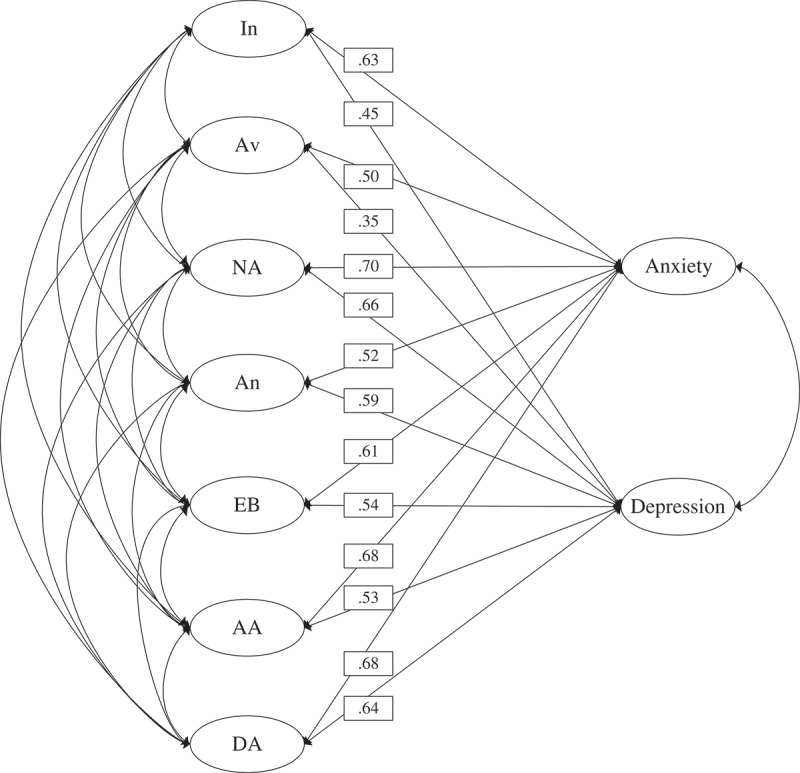
Structural model of the seven-factor PTSD model and the latent anxiety and depression factors. Note: *N* = 836. In = Intrusion; Av = Avoidance; NA = Negative affect; An = Anhedonia; EB = Externalizing behaviours; AA = Anxious arousal; DA = Dysphoric arousal. All correlations are statistically significant (*p* < .01).

The nine-factor model yielded an acceptable fit, indicated by χ^2^ (491, *N* = 836) = 1281.722, CFI = .922, TLI = .911, RMSEA = .044 (90% CI: .041–.047). Correlation coefficients between PTSD factors and external measures of anxiety and depression are presented in Figure 1. Results from Wald chi-square tests indicated that: intrusion, avoidance and anxious arousal factors correlated more strongly with the anxiety factor than with the depression factor (Wald χ^2^ (1, *N* = 836) = 40.27, *p* < .01, Wald χ^2^ (1, *N* = 836) = 26.48, *p* < .01, and Wald χ^2^ (1, *N* = 836) = 29.64, *p* < .01, respectively); the anhedonia factor correlated more strongly with the depression factor than with the anxiety factor (Wald χ^2^ (1, *N* = 836) = 7.05, *p* < .01); the negative affect and dysphoric arousal factors correlated with the anxiety and depression factors equivalently (Wald χ^2^ (1, *N* = 836) = 2.52, *p* = .112 and Wald χ^2^ (1, *N* = 836) = 2.04, *p* = .153, respectively).

## 4. Discussion

By using data yielded from a sample of adolescents suffering from an explosion accident, the present study investigated the latent dimensions of DSM-5 PTSD symptoms. Four competing models were first tested, including the current DSM-5 model, the anhedonia model, the externalizing behaviours model and a newly refined seven-factor hybrid model. CFA results indicated that the seven-factor hybrid model demonstrated superior fit relative to the other competing models. The results are generally congruent with prior findings with adolescent samples exposed to earthquake (Wang et al., 2015) and various traumatic events (Liu et al., 2016), and suggest that DSM-5 PTSD symptoms appeared in traumatized youths could be best captured by intrusion, avoidance, negative affect, anhedonia, externalizing behaviours, anxious arousal and dysphoric arousal factors. Considering that we utilized a sample exposed to an explosion accident, the systematic replication with victims suffering from different traumatic events provides and extends empirical support for the seven-factor hybrid model, and added to extant understanding on the complexity of youth’s responses to traumatic events.

As noted by researchers (e.g., Armour et al., 2016), a diagnostic model cannot be validated with internal fit statistic alone. By examining relationships between PTSD’s factors and two external psychopathological variables (anxiety and depression), this study further assessed the external convergent and discriminant validity of the seven-factor hybrid model. We constructed research hypotheses based on previous theoretical and empirical studies suggesting distinguishable relationships between PTSD’s factor and the anxiety and depression constructs (e.g., Elhai et al., 2011; Watson, 2009). The results showed that the anxiety-related PTSD constructs (i.e., intrusion, avoidance and anxious arousal) displayed higher correlation with external anxiety measure than with depression measure; the depression-related PTSD construct (i.e., anhedonia) displayed higher correlation with external depression measure than with anxiety measure; the both anxiety and depression-related PTSD constructs (i.e., negative affect and dysphoric arousal) displayed equivalent correlation with external anxiety and depression measures. The findings confirmed our research hypotheses, yielding further empirical evidence supporting the external convergent and discriminant validity of the newly refined seven-factor hybrid model.

This study has several clinical implications. First, as outlined earlier, the DSM-5 four-factor PTSD model is largely built on previous CFA studies on DSM-IV symptoms (Friedman, 2013). The theoretically and empirically supported seven-factor hybrid model extends current understanding of PTSD constructs, and informs further refinement of more sophisticated assessment and diagnostic procedure by ensuring we are measuring the ‘correct’ and relevant symptom domains. Second, the current findings also add to extant knowledge on the heterogeneity of human’s responses to traumatic stressors, and inform thinking about the place of this disorder within the nosology of psychiatric conditions. As PTSD is involving in anxiety-related, depression-related, and no-specific symptoms, and not exclusively a mood or anxiety disorder, it may be more appropriate to remove PTSD from anxiety disorders into the new ‘Trauma and Stressor-Related Disorders’. Third, this study also adds to the knowledge on comorbidity between PTSD and other mood and anxiety disorders. In this study, we found that negative affect and dysphoric arousal are not distinguishable in terms of their relations with anxiety and depression, which implies that the constructs are shared by PTSD, anxiety and depression, and may be non-specific and transdiagnostic symptoms contributing to high comorbidity of these disorders. According to several researchers (e.g., Spitzer, First, & Wakefield, 2007), these symptoms should be removed from the diagnostic criteria entirely for their lack of specificity to PTSD. However, subsequent empirical studies reported that removing these symptoms does not seem to affect comorbidity rates (e.g., Elhai, Grubaugh, Kashdan, & Frueh, 2008). Thus, it would not be wise to remove these symptoms, although the exact role of them within the diagnosis is not clear at this time.

Several limitations of this study should be acknowledged. First, the current findings were limited by our utilization of a convenience sample exposed to a particular traumatic event from China. Thus, the findings need to be further tested with representative samples exposed to a range of traumatic events from other cultural backgrounds. Second, this study used a non-clinical sample with very low prevalence of PTSD and self-reported measures. Therefore, further replications with clinical samples and clinician-rated measures are warranted. Third, only two external variables were adopted to evaluate the convergent and discriminant validity of the seven-factor hybrid model. To accumulate more solid empirical evidences for the model, further studies including a range of psychological, behavioural and biological variables are needed.

Despite the limitations, with a sample of adolescents exposed to an explosion accident, this study yielded further empirical evidence in favour of internal and external validity of the newly proposed seven-factor hybrid model of DSM-5 PTSD symptoms. The findings add to limited literature on latent dimensions of DSM-5 PTSD symptoms in traumatized youths, and have implications for further trauma-related clinical practice and research.

## References

[CIT0001] American Psychiatric Association (2013). *Diagnostic and statistical manual of mental disorders*.

[CIT0002] Armour C. (2015). The underlying dimensionality of PTSD in the diagnostic and statistical manual of mental disorders: Where are we going?. *European Journal of Psychotraumatology*.

[CIT0003] Armour C., Elhai J. D., Richardson D., Ractliffe K., Wang L., Elklit A. (2012). Assessing a five factor model of PTSD: Is dysphoric arousal a unique PTSD construct showing differential relationships with anxiety and depression?. *Journal of Anxiety Disorders*.

[CIT0004] Armour C., Műllerová J., Elhai J. D. (2016). A systematic literature review of PTSD’s latent structure in the diagnostic and statistical manual of mental disorders: DSM-IV to DSM-5. *Clinical Psychology Review*.

[CIT0005] Armour C., Tsai J., Durham T. A., Charak R., Biehn T. L., Elhai J. D., Pietrzak R. H. (2015). Dimensional structure of DSM-5 posttraumatic stress symptoms: Support for a hybrid anhedonia and externalizing behaviors model. *Journal of Psychiatric Research*.

[CIT0006] Bovin M. J., Marx B. P., Weathers F. W., Gallagher M. W., Rodriguez P., Schnurr P. P., Keane T. M. (2016). Psychometric properties of the PTSD checklist for diagnostic and statistical manual of mental disorders–fifth edition (PCL-5) in veterans. *Psychological Assessment*.

[CIT0007] Cuthbert B. N. (2014). The RDoC framework: Facilitating transition from ICD/DSM to dimensional approaches that integrate neuroscience and psychopathology. *World Psychiatry*.

[CIT0008] Elhai J. D., Biehn T. L., Armour C., Klopper J. J., Frueh B. C., Palmieri P. A. (2011). Evidence for a unique PTSD construct represented by PTSD’s D1-D3 symptoms. *Journal of Anxiety Disorders*.

[CIT0009] Elhai J. D., Grubaugh A. L., Kashdan T. B., Frueh B. C. (2008). Empirical examination of a proposed refinement to DSM-IV posttraumatic stress disorder symptom criteria using the National Comorbidity Survey Replication data. *Journal of Clinical Psychiatry*.

[CIT0010] Friedman M. J. (2013). Finalizing PTSD in DSM-5: Getting here from there and where to go next. *Journal of Traumatic Stress*.

[CIT0011] Helpman L., Rachamim L., Aderka I. M., Gabai-Daie A., Schindel-Allon I., Gilboa-Schechtman E. (2015). Posttraumatic symptom structure across age groups. *Journal of Clinical Child & Adolescent Psychology*.

[CIT0012] Kelley L. P., Weathers F. W., McDevitt-Murphy M. E., Eakin D. E., Flood A. M. (2009). A comparison of PTSD symptom patterns in three types of civilian trauma. *Journal of Traumatic Stress*.

[CIT0013] Kline R. (2011). *Principles and practice of structural equation modeling*.

[CIT0014] Liu L., Wang L., Cao C., Qing Y., Armour C. (2016). Testing the dimensional structure of DSM-5 posttraumatic stress disorder symptoms in a nonclinical trauma-exposed adolescent sample. *Journal of Child Psychology and Psychiatry*.

[CIT0015] Liu P., Wang L., Cao C., Wang R., Zhang J., Zhang B., Elhai J. D. (2014). The underlying dimensions of DSM-5 posttraumatic stress disorder symptoms in an epidemiological sample of Chinese earthquake survivors. *Journal of Anxiety Disorders*.

[CIT0016] Lovibond P. F., Lovibond S. H. (1995). *Manual for the depression anxiety stress scales*.

[CIT0017] Mordeno I. G., Carpio J. G. E., Nalipay M. J. N., Saavedra R. L. J. (in press). PTSD’s underlying dimensions in typhoon Haiyan survivors: Assessing DSM-5 symptomatology-based PTSD models and their relation to posttraumatic cognition. *Psychiatric Quarterly*.

[CIT0018] Raftery A. E. (1995). Bayesian model selection in social research. *Sociological Methodology*.

[CIT0019] Seligowski A. V., Orcutt H. K. (2016). Support for the 7-factor hybrid model of PTSD in a community sample. *Psychological Trauma: Theory, Research, Practice, and Policy*.

[CIT0020] Spitzer R. L., First M. B., Wakefield J. C. (2007). Saving PTSD from itself in DSM-V. *Journal of Anxiety Disorders*.

[CIT0021] Szabó M. (2010). The short version of the Depression Anxiety Stress Scales (DASS-21): Factor structure in a young adolescent sample. *Journal of Adolescence*.

[CIT0022] Tsai J., Harpaz-Rotem I., Armour C., Southwick S. M., Krystal J. H., Pietrzak R. H. (2015). Dimensional structure of DSM-5 posttraumatic stress disorder symptoms: Results from the National Health and Resilience in Veterans Study. *Journal of Clinical Psychiatry*.

[CIT0023] Wang K., Shi H., Geng F., Zou L., Tan S., Wang Y., Chan R. C. K. (2016). Cross-cultural validation of the depression anxiety stress scale–21 in China. *Psychological Assessment*.

[CIT0024] Wang L., Zhang L., Armour C., Cao C., Qing Y., Zhang J., Fan G. (2015). Assessing the underlying dimensionality of DSM-5 PTSD symptoms in Chinese adolescents surviving the 2008 Wenchuan earthquake. *Journal of Anxiety Disorders*.

[CIT0025] Wang R., Wang L., Li Z., Cao C., Shi Z., Zhang J. (2013). Latent structure of posttraumatic stress disorder symptoms in an adolescent sample one month after an earthquake. *Journal of Adolescence*.

[CIT0026] Watson D. (2009). Differentiating the mood and anxiety disorders: A quadripartite model. *Annual Review of Clinical Psychology*.

[CIT0027] Weathers F. W. (2013). *The PTSD Checklist for DSM-5 (PCL-5): Development and initial psychometric analysis*.

[CIT0028] Wirth R. J., Edwards M. C. (2007). Item factor analysis: Current approaches and future directions. *Psychological Methods*.

